# Dr. Chauncey S. Lamb

**Published:** 1916-05

**Authors:** 


**Dr. Chauncey S. Lamb,** Buffalo 1893, died at his home in New Haven, March 23, aged 44.

